# Step Detection in Single-Molecule Real Time Trajectories Embedded in Correlated Noise

**DOI:** 10.1371/journal.pone.0059279

**Published:** 2013-03-22

**Authors:** Srikesh G. Arunajadai, Wei Cheng

**Affiliations:** 1 Department of Biostatistics, Columbia University, New York, New York, United States of America; 2 Department of Pharmaceutical Sciences, College of Pharmacy, University of Michigan, Ann Arbor, Michigan, United States of America; Oak Ridge National Laboratory, United States of America

## Abstract

Single-molecule real time trajectories are embedded in high noise. To extract kinetic or dynamic information of the molecules from these trajectories often requires idealization of the data in steps and dwells. One major premise behind the existing single-molecule data analysis algorithms is the Gaussian ‘white’ noise, which displays no correlation in time and whose amplitude is independent on data sampling frequency. This so-called ‘white’ noise is widely assumed but its validity has not been critically evaluated. We show that correlated noise exists in single-molecule real time trajectories collected from optical tweezers. The assumption of white noise during analysis of these data can lead to serious over- or underestimation of the number of steps depending on the algorithms employed. We present a statistical method that quantitatively evaluates the structure of the underlying noise, takes the noise structure into account, and identifies steps and dwells in a single-molecule trajectory. Unlike existing data analysis algorithms, this method uses Generalized Least Squares (GLS) to detect steps and dwells. Under the GLS framework, the optimal number of steps is chosen using model selection criteria such as Bayesian Information Criterion (BIC). Comparison with existing step detection algorithms showed that this GLS method can detect step locations with highest accuracy in the presence of correlated noise. Because this method is automated, and directly works with high bandwidth data without pre-filtering or assumption of Gaussian noise, it may be broadly useful for analysis of single-molecule real time trajectories.

## Introduction

The advent of single-molecule techniques [1], [2], [3], [4], [5], [6], [7] in recent years brought many interesting discoveries in chemistry, physics, and life sciences. One unique advantage of single-molecule technique is the ability to measure molecular processes in a heterogeneous environment without the need of synchronizing these molecules, and to unveil the static and dynamic disorders among individual molecules [1]. One broad class of single-molecule measurement is movement of molecular motors in real time. These molecular motors move in steps [8], [9], [10], [11], [12]. Statistics on their movement trajectories can reveal rich mechanistic information that is often inaccessible from conventional bulk experiments.

Different types of statistical tools have been developed for analysis of these data to extract characteristics of motor movement. For stepping of molecular motors that can be observed directly from time trajectories, pairwise distance distribution analysis was among the first to be used for this task [13]. A Fourier analysis of the pairwise distance distribution histogram can reveal the periodicity in single-molecule trajectories, which is an objective measure of motor step size. Application of this method to different molecular motors has revealed their apparent step sizes of movement [11], [14], [15], [16], [17], although this analysis does not yield information on the dwell time in between motor steps, which is essential in deducing the coupling of fuel molecule to motor movement. To this end, algorithms for detection of both steps and dwells have been developed by investigators [18], [19], [20], [21], [22], [23], [24], [25], and the performance of several methods has been quantitatively compared [26]. In particular, the algorithm developed by Kerssemakers et al. [19] (referred as KERS herein) has found increasing use in different motor systems [27], [28], [29]. In this method, the original data was assumed to be a step function buried in Gaussian noise. The motor steps are found in successive iterations: the plateaus of the steps identified in a previous cycle are further divided to find additional steps. The quality of the fit was assessed using a statistic S, which is the ratio between the Chi-squared of a counter fit and the Chi-squared of the best fit. For molecular motors that can be measured at single-molecule level but whose individual steps are obscured by measurement noise, techniques have also been developed to extract step size information from variance in long trajectories of motor movement [30]. Under these circumstances, even though the individual steps of the motor cannot be identified directly from time traces [31], [32], [33], estimation of motor step size using this technique has yielded values that are comparable to results from other complementary approaches [34].

Despite the diversity of these different step-detection algorithms, a common practice is the assumption of Gaussian white noise in the experimental data, which is independently distributed and shows no correlation with regard to time. This assumption may be true in certain cases, but has not been thoroughly validated in general. Any noise that has frequency-dependent amplitude will deviate from Gaussian white noise. This so-called ‘colored’ noise displays autocorrelations and widely exists in nature [35]. For example, colored noise is typically present in lasers that are used to form optical tweezers. Both intensity and pointing stability of the laser display noise whose amplitudes depend on bandwidth [36], [37]. As we show, colored noise is present in single-molecule real time trajectories collected from optical tweezers. The assumption of Gaussian white noise for single-molecule data that contains colored noise can result in significant fitting errors. It is thus critical to assess the structure of the noise when analyzing these single-molecule trajectories. We have developed a statistical step detection algorithm based on Generalized Least Squares (referred as GLS herein) that explicitly takes the structure of the noise into account. This algorithm allows one to identify motor steps and dwells directly from time trajectories in the presence of highly autocorrelated noise and provides standard errors and confidence intervals associated with these steps. There is no assumption on a single unique step size in this algorithm. Indeed, variation in size of steps can be fully taken into account [38]. There is no requirement on the motor to be highly processive [30]. The time trajectory can still be analyzed even though the motor can detach from its track prematurely. We present this method in detail and compare it with the KERS method. As we demonstrate, this GLS method can detect steps with highest accuracy in the presence of correlated noise, which can significantly minimize errors in data analysis and interpretation. Because this GLS method can work with high bandwidth data directly without any pre-filtering, it may be broadly applicable to single-molecule data analysis in general.

## Results and Discussion

### Correlated Noise in Single-molecule Trajectories

**Figure 1:**
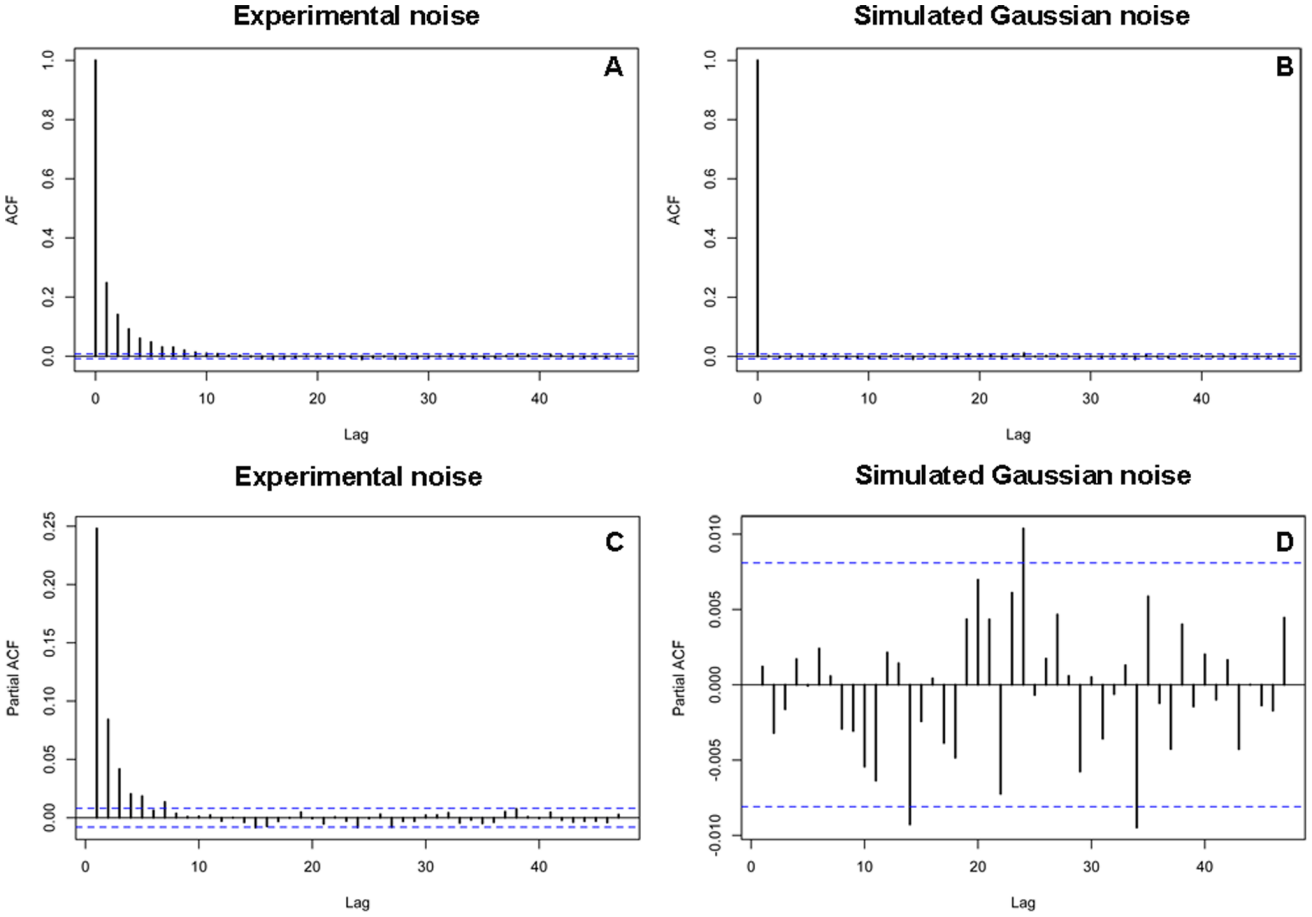
Correlated noise in single-molecule real time trajectories. The autocorrelation function (ACF) and the partial autocorrelation function (PACF) for AR noise of order 7 as observed in a typical RNA unwinding trace (A and C). The plots from simulated Gaussian noise were also shown for comparison (B and D). The horizontal lines indicate the 95% confidence intervals under the null hypothesis of no correlation. For the AR(7) noise, the ACF shows exponentially decay while the PACF gradually cuts off, i.e. goes to zero after lag 7. For Gaussian noise, the ACF is 1 at lag 0 and zero for other lags while the PACF is zero for all lags.

The structure of noise in a real time trajectory can be revealed by calculating the autocorrelation function (ACF) of the data. Gaussian white noise will display a delta function while autocorrelated noise will show an exponential decay for its ACF. We have extensively computed the ACF for real time single-molecule trajectories collected with high resolution optical tweezers [38]. A typical result is shown in Fig. 1A, which shows a clear exponential decay. In contrast, a simulated Gaussian white noise shows the expected delta function (Fig. 1B). This result demonstrates that the experimental single-molecule trajectory indeed contains correlated noise, i.e., the noise amplitude at the current moment is a function of past noise and some random error, which induces a correlation structure in the noise. The order of this correlation structure can be further assessed using the plot of partial autocorrelation function (PACF) [39]. Gaussian white noise will display zero everywhere throughout the PACF while for autocorrelated noise of order p, the PACF is zero for lags greater than p and non-zero otherwise [39]. As shown in Fig. 1C, the corresponding PACF of Fig. 1A shows non-zero amplitude before lag 7 and zero thereafter, highlighted by the horizontal dashed lines that indicate the 95% confidence intervals under the null hypothesis of no correlation, thereby suggesting an order 7 for this noise, i.e., the noise is a function of past seven values of noise and some random error. In contrast, the simulated Gaussian noise has zero amplitudes everywhere throughout the PACF (Fig. 1D).

### Step Detection in the Presence of Correlated Noise

The above results show that experimental single-molecule trajectories contain noise that is correlated in time. Would it still be fine to assume Gaussian white noise when we analyze these traces? To address this question, we have developed a step detection method using GLS (Materials and Methods). In this method, we have the option of assuming Gaussian white noise for the data to be analyzed, or explicitly take the structure of the noise into account based on PACF analysis. To examine the impact of Gaussian noise assumption on data analysis, we generated simulated single-molecule trajectories embedded in autocorrelated noise, and compared step detection with and without Gaussian assumption for the added noise. In addition, we also analyzed the same set of traces using KERS method in order to compare with the GLS method. Fig. S1A shows a simulated step function that resembles real time RNA unwinding traces based on our recent publication [38]. It consists of a series of upward steps that are occasionally interrupted by downward steps. We represent the time axis by indexing integers for easy identification. We then added noise to the step function to generate mock unwinding traces. One such realization is shown in Fig. S1B. The noise is simulated from an autoregressive process of order 7 (Fig. 1C), with coefficients 0.222, 0.072, 0.035, 0.015, 0.016, 0.003 and 0.013 that are typically found from the published single-molecule trajectories [38]. We independently simulated the noise 100 times to generate 100 mock traces. We then use three different procedures to identify steps and dwells in these traces and compare them: (1) the GLS method; (2) exactly the same procedure as GLS method but ignoring autocorrelation in the noise, i.e., assuming Gaussian white noise even though the added noise is correlated; and (3) the KERS method.

**Figure 2:**
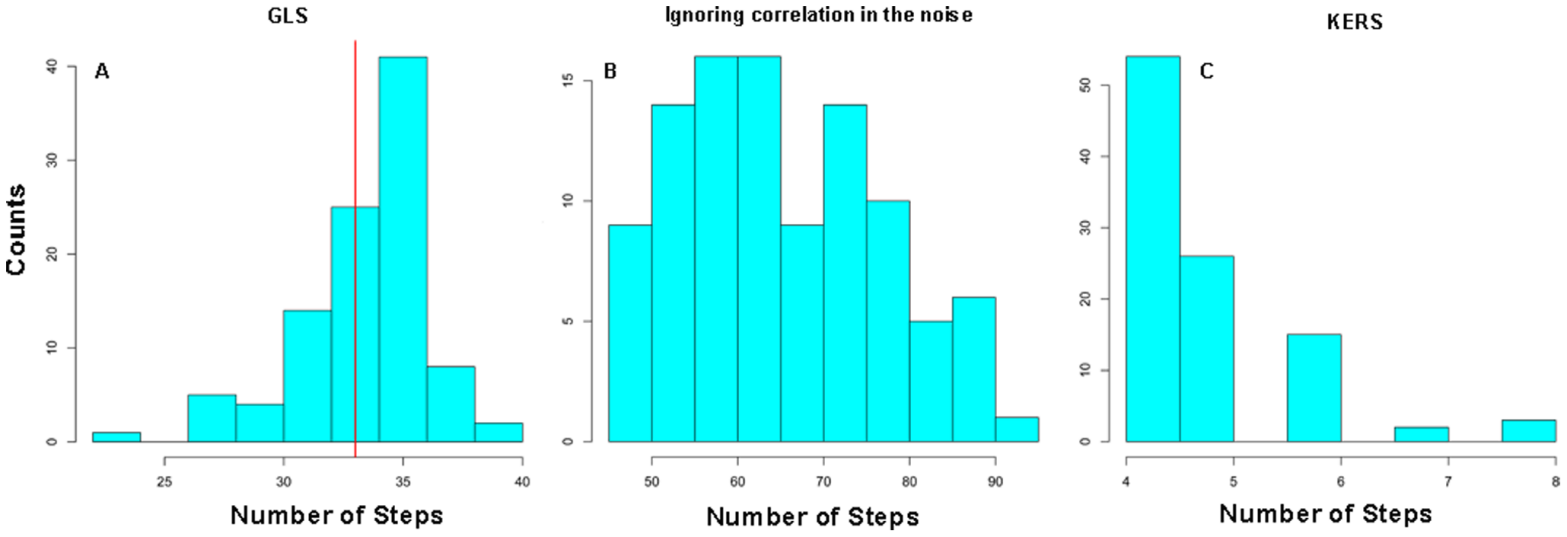
Histogram of the number of steps detected from 100 realizations of the simulated traces. (A), (B) and (C) show the results from GLS method, GLS method but ignoring the correlation in the noise and KERS method, respectively.

**Figure 3:**
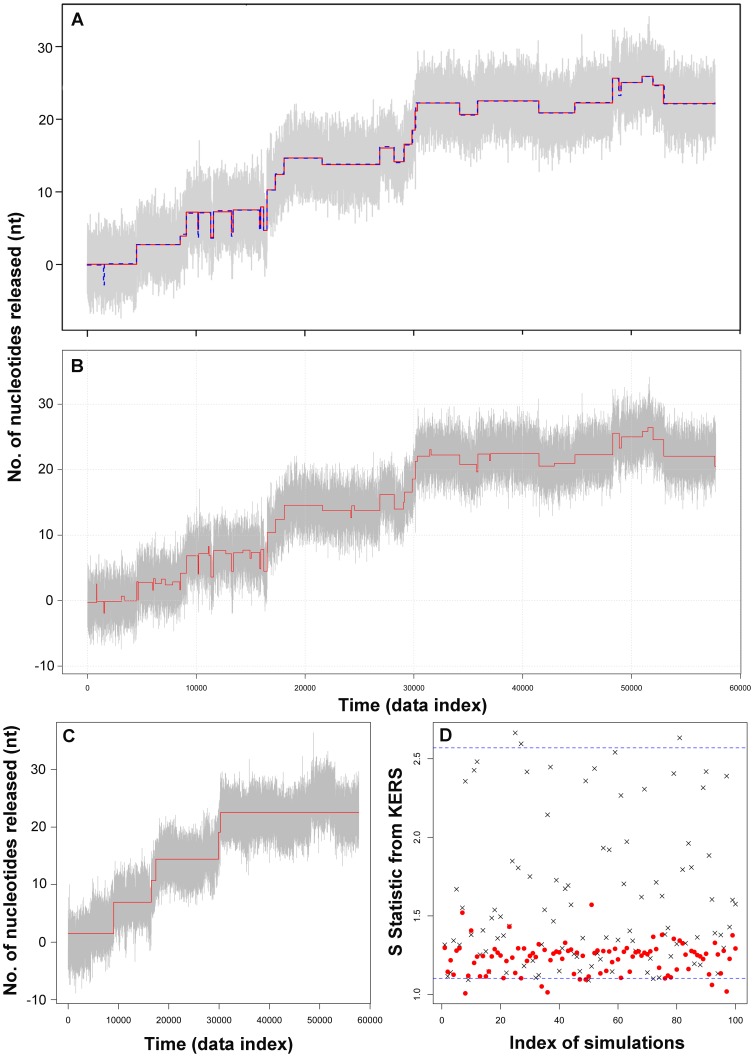
Over- and underestimates of step numbers in the test simulated traces. (A) A representative best fit by the GLS method for data that contains correlated noise; the 2.5 kHz test trace is shown in grey and the fit is shown in red. The original step function is shown in blue dashed line for comparison. (B) One of the best fits obtained from GLS method by ignoring correlation for data that contains correlated noise; the 2.5 kHz test trace is shown in grey and the fit is shown in red. (C) and (D) Fit and S-Statistic distribution from KERS method. (C) One of the best fits from KERS method; the 2.5 kHz test trace shown in grey and the fit is shown in red. (D) Distribution of S-statistic as a result of fitting using KERS method. The crosses are the S-statistic from the known step function and the red dots from the best fit for each trace.

Fig. 2 shows the histograms of the number of steps detected from the above procedures. As listed in Table 1 and shown in Fig. 2A, the GLS method on average detected 34 steps from these traces, ranging between 23 and 40, which compares very well with the total number of 33 steps in the simulated step function. Fig. 3A shows a representative best fit (red line) from this procedure, which shows close resemblance to the original step function (blue dashed line). Repeating the same procedure but ignoring autocorrelation in the noise vastly overestimates the number of steps, with a mean of 66 steps, ranging between 45 and 91 (Fig. 2B). Fig. 3B shows a representative best fit from this second procedure. Comparison between Fig. 3B and Fig. S1 suggests that majorities of the steps in the simulated trace were identified, but a significant fraction of these steps are false positives, because they do not exist in the original trace. *These false positives were identified as a result of the autocorrelated noise*, *which was not accounted for in this step detection procedure*. To confirm the impact of this correlated noise on step detection, we used the same step function as shown in Fig. S1, but added Gaussian white noise, and repeated the same step detection procedure. Fig. S2A shows a representative best fit from this procedure. Interestingly, it now detects correct number of steps on average (Table 1). These results demonstrate that the structure of the underlying noise in a single-molecule trajectory has a profound impact on the outcome of step detection. The assumption of Gaussian white noise on otherwise correlated noise can lead to a significant overestimation for the number of steps in a trace.

**Table 1:** Summary of number of steps detected from 100 realizations of the simulated traces.

	Min.	1^st^ Qu.	Median	Mean	3^rd^ Qu.	Max.
(a) GLS	23.00	33.00	35.00	33.79	36.00	40.00
(b) Ignoringcorrelation	45.00	56.00	63.50	65.64	75.00	91.00
(c) True Gaussian	26.00	28.00	30.00	29.75	31.00	36.00
(d) KERS	4.00	4.00	4.00	4.74	5.00	8.00

Four different procedures were used to detect steps, where Procedure (a), (b) and (d) are for traces with correlated noise analyzed with GLS method (a), ignoring noise correlation and assuming Gaussian noise (b) and the KERS method (d); Procedure (c) was done for traces with Gaussian white noise that were analyzed using the GLS method.

In contrast to the second procedure, the KERS method vastly underestimates the number of steps, with a mean of 5 steps and a range between 4 and 8 (Fig. 2C). Fig. 3C shows a representative best fit from KERS method. Fig. 3D shows the S-statistic obtained throughout the 100 mock traces. For each realization, the S-statistic from the original step function (true S value) was shown as crosses and that from the best attempted fit was shown as red dots. Although the true S values are generally higher than those from the best attempted fits (indicating a better fit), the S-statistic from the attempted fits are within random variable limits of the true S value as indicated by the horizontal 95% confidence interval lines. This result suggests that the KERS method can give results that deviate significantly from reality. One possible reason behind this is the high bandwidth of the data (2.5 kHz). To test this, we used a boxcar filter with a window size of 10 to filter and decimate the trace, and attempted again with KERS method. A representative result is shown in Fig. S2B. It now detects 11 steps instead of 4, closer to reality but still much lower than the true value of 33. This result suggests that the KERS method is highly dependent on the bandwidth of the data, and was not able to correctly identify the steps in the original trace even after filtering. As a result, the clear advantage of GLS method is that it can work with high bandwidth data directly without any filtering. In summary, noise structure should be accounted for in single-molecule data analysis. Assumption of Gaussian white noise can lead to either over- or underestimation of the number of steps depending on the algorithms used. Moreover, the GLS method outperforms the KERS method (which assumes Gaussian white noise) and on average detects the correct number of steps.

**Figure 4:**
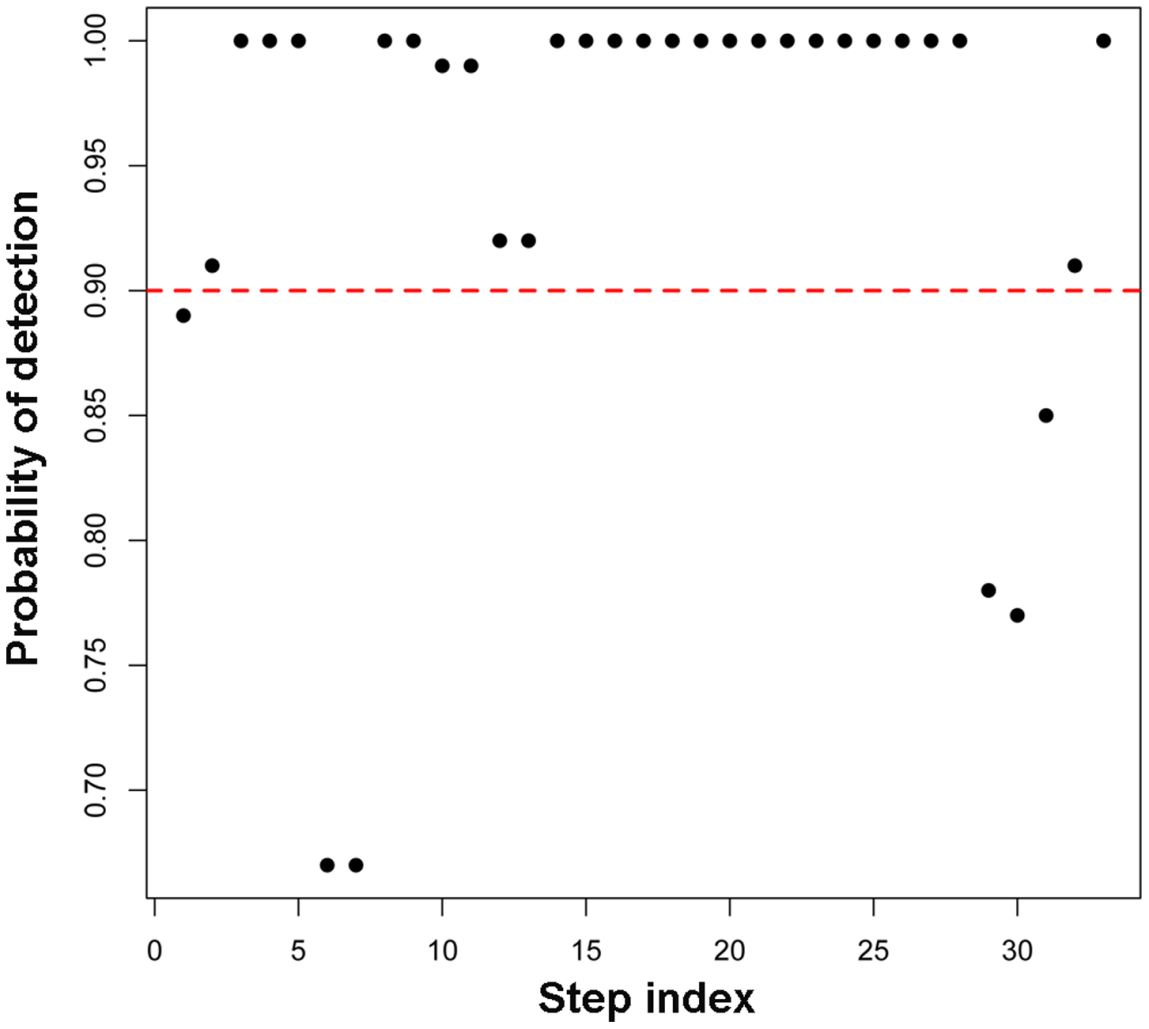
Efficiency of step detection using GLS method. The proportion of the traces in which a given step was detected was plotted as a function of the step index. The red dashed line indicates 90%.

Despite being the best among the three procedures, results shown in Fig. 2A and Table 1 indicate that the GLS method can still over- or underestimate the number of steps in a trace. To examine these deviations in more detail, Table 2 shows the statistics of false positives (non-existing steps but detected as a step) and true negatives (true steps that were not detected) from the mock traces analyzed with the GLS method. On average, the median of false positives was 1. The median of true negatives was zero with 75% of the traces having at most 2 true negatives. This result suggests that GLS does a very good job in identifying almost all the steps that are present, although it can occasionally detect false positives. Fig. 4 further shows the fraction of traces in which a given step was detected. Over the total 33 simulated steps, 70% of them were detected every time by GLS. The steps whose detection efficiency drops below 90% (indicated by the red dashed line) are usually the transient steps, i.e., those steps that have very short dwell times in between, as seen from Fig. S1B. Specifically, these are steps # 1 (31 ms), # 6 (18 ms), # 7(18 ms), # 29(80 ms), # 30(80 ms) and # 31(84 ms), where the dwell times in milliseconds are noted in parentheses. These dwell times are relatively short in comparison to other dwells, which ranged between 0.1 and 2.25 s. Still, the detection efficiency for all these transient events is greater than 65%, which is in contrast to KERS method where detection of transient steps depends on filtering and is below 50% even for filtered data (Fig. S2B). This is a very important feature of GLS method, because one of the distinct advantages of single-molecule real time measurement is to reveal transient events. *If step detection requires data filtering, then these transient events are likely to be masked as a result of filtering*.

**Table 2:** Summary of false positive and true negative steps from 100 realizations of the simulated traces.

	Min.	1^st^ Qu.	Median	Mean	3^rd^ Qu.	Max.
False positives	0.00	1.00	1.00	1.52	2.00	6.00
True negatives	0.00	0.00	0.00	1.63	2.00	8.00

The traces contained correlated noise and the steps were identified using GLS method to take the noise structure explicitly into account.

**Figure 5:**
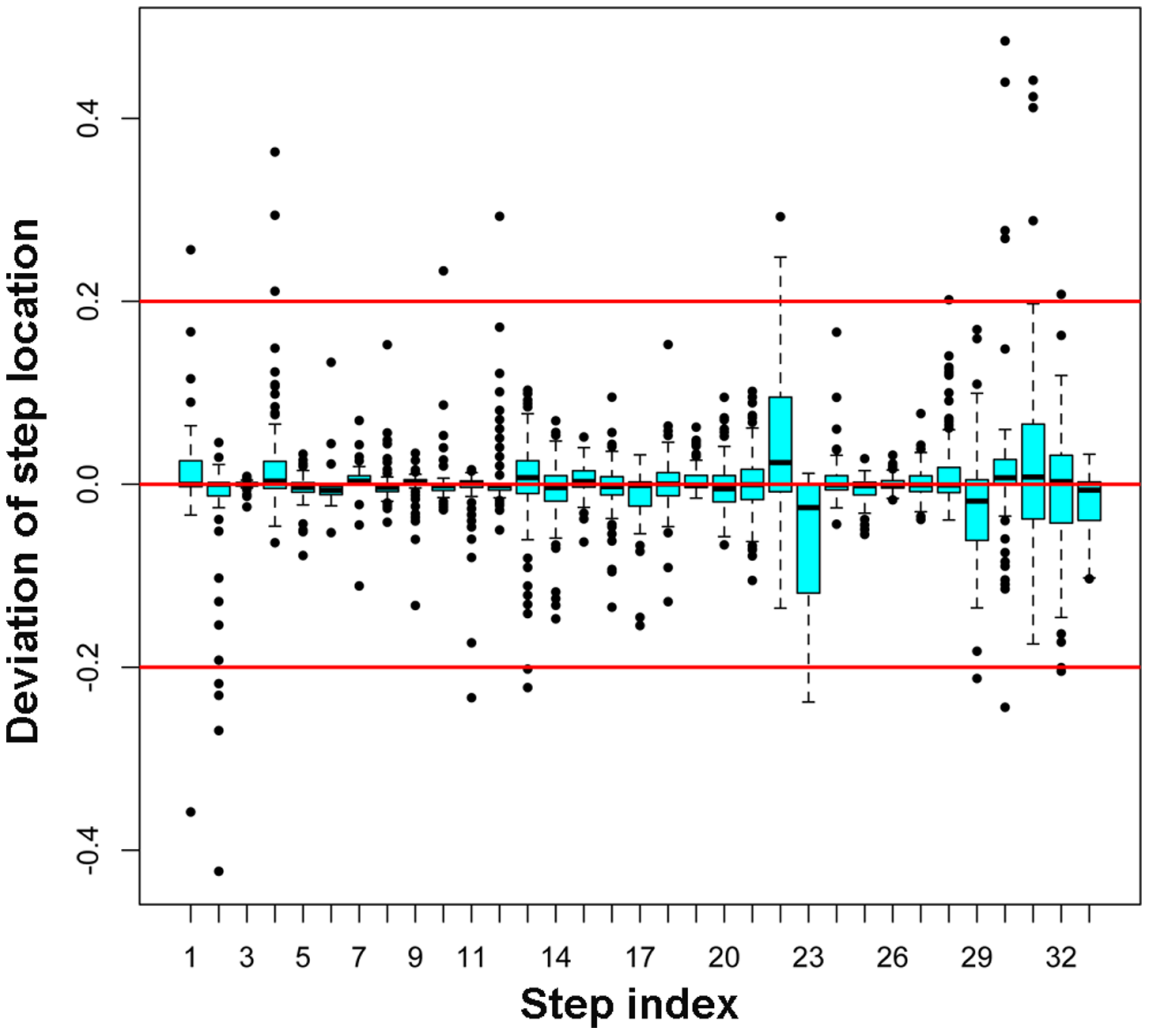
Precision of identified step location using GLS method. Deviation of a step from its true location as a percentage of the plateau length was plotted as a function of the step index in a box plot. The cyan boxes indicate the middle 75% of the data or the interquartile range (IQR). The extended lines or whiskers mark the 1.5×IQR distance. Any point greater or less than this value is an outlier and are shown by the dots.

The duration of dwells in between steps are of significant interest in single-molecule real time trajectories. These dwells are computed as the time elapsed between two steps. Typically, these dwells represent the waiting time the motor has to take before next motion, which is usually coupled to fuel binding under limiting fuel concentrations. It is therefore important to quantitate the accuracy with which a step location can be identified. To this end, we first detected steps using GLS method from the set of test traces. We then quantified the deviation of the identified step location from its true step location. This deviation is computed as the difference in time (data index) between the two, which is further normalized by the lengths of the true dwell time before and after that step (Fig. 5). For example, imagine the dwell to the left of a true step location be of length 50 and to the right be length 100. If the step is identified 10 points to the left of the true location we indicate its deviation as −20%; if the step is identified at the exact location then it is 0%; and if it is identified 30 points to the right of the true step location, we indicate it as +30%. Fig. 5 shows the percentage of deviation for each step as computed above from GLS method. It can be seen that for most of the steps, the step deviation is close to zero on average. The majorities of the deviations are within ±20% of the true step locations, as indicated by the red solid lines.

In summary, the GLS method can efficiently identify almost all the steps in a single-molecule trajectory, and the step locations were identified with very good precision. Recently, the exact same method has been applied to the single-molecule unzipping trajectories of the hepatitis C virus NS3 RNA helicase [38]. The advantages of this method to work with high bandwidth raw data without any pre-filtering or assumption of Gaussian noise, and its ability to detect transient steps are likely to be useful for single-molecule real time data analysis in general.

## Materials and Methods

### Partial Autocorrelation Function (PACF)

Compared to ACF, the PACF for a time series is the correlation between time lags after removing the effects of the intermediary points. Please see Supporting Information for further details.

### Step Detection Framework using GLS

For a single-molecule real time trajectory measured from time t = 0 to t = T, we denote the times at which the k steps of the trajectory occur with t_j_, j = 1,…,k. The steps in a trace can be set up as a regression function given by
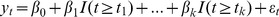
(1)where y_t_, t = 0,…, T is the observed trace; I(t ≥ t_j_; j = 1,…,k) are indicator functions such that I(t ≥t_j_) = 1 and 0 otherwise (similar to the Heaviside step function). ε_t_ is the underlying noise. Here β_0_ is the baseline at which the trace begins at t = 0 and β_j_, j = 1,…, k are the step sizes of the k steps, with a negative value indicating a downward step.

In general, ε_t_ is assumed to be independent and identically distributed (i.i.d) as zero-mean Gaussian noise N(0, σ^2^) with variance σ^2^ [18], [19], [20], [21], [22], [23], [26], in which case one can obtain least squares estimates (LSE) for the parameters β_j_, j = 0,…, k. Let Θ = {β_0_,…, β_k_; σ^2^} denote the vector of parameters to be estimated. Let f_Θ_(•) denote the density function of the error term ε_t_ dependent on the parameter vector Θ. The parameter Θ, may be estimated by maximizing the likelihood function given by
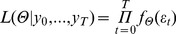
(2)or equivalently the log-likelihood function given by



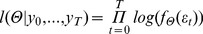
(3)Estimates obtained using Eq. 2 or 3 is referred to as maximum likelihood estimates (MLE). In the case of i.i.d Gaussian noise, LSE and MLE are identical. As the number of parameters in Θ increases, i.e. the number of steps increases, the likelihood *L* or *l* will increase (or equivalently in the Gaussian case, the residual sum of squares will decrease). This can create a tendency to overfit, i.e., more steps can always produce a better fit than less. To avoid over-fitting the trace we present two criteria to choose the optimal number of steps. The Akaike Information Criterion (AIC) given by
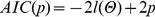
(4)and Bayesian Information Criterion (BIC) given by
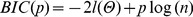
(5)where p is the total number of parameters to be estimated in the model, n is the number of observations and l(Θ) is the log-likelihood function given in Eq. 3. As the negative log-likelihood decreases with increasing number of parameters, both AIC and BIC penalize by the number of parameters in the model. For n >7, the BIC offers a higher penalty to the model. The model with the least AIC or BIC value is chosen as the optimal model.

So far we have assumed that the underlying noise ε_t_ is i.i.d Gaussian. This assumption may be violated, as shown in Fig. 1 from single-molecule traces collected with high-resolution optical tweezers. In such cases, the noise is autocorrelated. A general procedure to model autocorrelated noise is to use an autoregressive (AR) noise of order p [39], given by
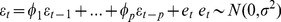
(6)i.e. the noise is a function of p past values of the noise and a random error, which induces correlation in the noise. We assume that AR noise is second order stationary, i.e., the mean is constant (zero) and the correlation between any two time points is dependent only on the lag h between them and not on the absolute time.

In the presence of autocorrelated noise, least squares can be expected to give unbiased estimates of the parameters but will not be efficient, i.e., parameters will have higher variances amongst all unbiased estimators unless ε*_t_* is uncorrelated with constant variance [40], [41]. Thus the estimates are not suitable for purposes of inference. To find the optimal solution in the presence of autocorrelated noise, one resorts to GLS [42]. To realize GLS efficiently, we rewrite Eq. 6 as
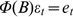
(7)where
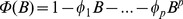
(8)and B is the backward shift operator such that B^p^ε_t_ = ε_t-p_. Applying the filter given by Eq. 8 to Eq. 1, we get



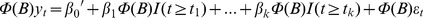
(9)

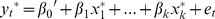
(10)


One can see from Eq. 10 that the error term is now i.i.d Gaussian and we can estimate the parameters of the model as before using this transformed equation. This transformation procedure, referred to as the Cochrane-Orcutt scheme [43], is computationally feasible for long time series as in high bandwidth single-molecule data.

Based on the above framework, one needs to know the order of the AR noise p in order to estimate the step size. The order p is determined and the corresponding coefficients are estimated as part of the GLS procedure. First, the steps are fitted assuming i.i.d Gaussian noise. The resulting residuals are examined for any autocorrelation. If the noise is indeed i.i.d Gaussian, then no further steps are required. If the noise is autocorrelated, then the order and coefficients are determined for the noise using standard time series estimation techniques [39]. Having estimated p and the coefficients, the step sizes are re-estimated using the Cochrane-Orcutt scheme described above and the BIC value associated with the fit is computed. After each round of fitting, we used student t-test to compute the p-value for each step and removed the step with the largest p-values from each fitting process. This process is then repeated until no further steps are left, and thus generated a series of fits with different BIC values for the original trajectory. The fit with the lowest BIC among all was chosen as the final model.

### Obtaining a Superset of Plausible Step Locations

To implement the above GLS procedure, one requires a set of plausible step locations to start with, from which an optimal number of steps can be chosen to fit the experimental trajectory. To this end, we developed a statistic η to generate the superset of plausible step locations as follows.

We represent each of the data point in the trace as y_i_ (i = 1,…,n). Consider a window of size 2w+1 centered at the data point, i.e., there are w data points on either side of the given point. We represent this window using the vector y_i,w_ = {y_i-w_,…, y_i_,…, y_i+w_}. At the ends of the series with indices less than w or greater than n-w, w is set to i-1 and n-i-1 respectively. Let y_i,w_^(min)^ and y_i,w_^(max)^ denote the minimum and maximum of the data points in y_i,w_, then R_i,w_ = y_i,w_^(max)^–y_i,w_^(min)^ denotes the range of the points within this window. Now consider the two halves of the window, the left half y_i,l_ = {y_i-w_,…, y_i_} and the right half y_i,r_ = {y_i_,…, y_i+w_}. Let q_i,l_ and q_i,r_ denote the vectors comprising the 0.25, 0.5 and 0.75^th^ quantile of the data in y_i,l_ and y_i,r_ respectively. We form the statistic
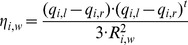
(11)where t denotes the transpose of the row vector. η_i,w_ is thus the mean squared difference of the quartiles on either side of the point i normalized by the square of the range R_i,w_. Normalization provides an upper bound of 1 for η_i,w_. If the distribution of the points on either side of i is identical then one would expect η_i,w_ to be close to zero. Conversely, if the distribution on either side of i is different, η_i,w_ is expected to be greater than zero. At step locations where the difference in the distributions on either side of the step point might be the greatest, one would expect the statistic η to increase to a local maximum right at the step location and decrease thereafter, forming local peaks around the step. The advantage of this statistic as compared to others is its sensitivity to changes in the overall shape and distribution of the data, i.e., the use of quartile that includes both the center and tail regions of the data points *instead of a single mean value used in the popular t-test*. Fig. S3A shows the value of the statistic η for all the points in the simulated trace shown in Fig. S1B using w = 500. The peaks can thus be considered as possible locations of motor steps. The choice of a window size is important. In reality, if the window size is too big, the variation in η will be smoothed out and one may miss the peaks corresponding to motor steps. To avoid this problem, we have used a set of windows of varying size, which range from 10 to 100 in steps of 10 and from 100 to 1000 in steps of 25, thus essentially make this procedure insensitive to data bandwidth and no need to filter data before analysis. Furthermore, a cutoff threshold, either 0.90^th^ or 0.95^th^ quantile of η was adopted. Only data points with η above the cutoff are considered in the superset of plausible steps. This procedure is adopted mainly to reduce computational burden. In practice, this threshold can be changed by the user. The lower the threshold, the greater the number of points chosen and thus greater computational burden. Fig. S3B shows the value of η from various windows (only a subset of the windows plotted for clarity of display) stacked on top of each other. The peaks chosen in each w using a cutoff threshold of 0.9 are highlighted by the red dots, which constitute the superset of plausible change points **C_L_**. Because majority of the red dots in Fig. S3B identify the same point, the number of points included in the final **C_L_** is much less than the total number of red dots in the figure.

The entire GLS algorithm was coded using ‘R’ [44], the statistical package that is freely available for download (http://www.r-project.org/). The R code together with instructions on how to run the algorithm to detect steps in real time single-molecule trajectory is freely available upon request.

## Supporting Information

Figure S1 **Simulated single-molecule RNA unwinding trajectory.** Panel (A) shows the simulated step function, which indicates the true underlying steps; (B) shows one realization of simulated unwinding trace after addition of AR noise of order 7 on top of the step function shown in (A). The step function is shown in red, and the trajectory with noise is shown in grey.
(TIF)

Figure S2 **Representative best fits of simulated RNA unwinding traces from two different procedures.** Panel (A) shows one of the best fits obtained from GLS method. The trajectory was simulated from the step function shown in Fig. S1A plus Gaussian white noise. The fit is in red, and the trajectory at 2.5 kHz is in grey. Panel (B) shows one of the best fits for simulated trajectories obtained from KERS method. The trajectory was simulated from the step function shown in Fig. S1A plus correlated noise of AR(7), and further filtered and decimated to 250 Hz using a boxcar filter. The fit is in red, and the trajectory at 250 Hz is in grey.
(TIF)

Figure S3 **The statistic η computed for the simulated single-molecule trace in Fig. S1B.** (A) from a window size of 500 and (B) shows a stack of η calculated using a set of window size, which includes 10, 30, 40, 50, 60, 70, 80, 90, 100, 125, 200, 275, 350, 425, 500, 575, 650, 725, 800, 875, and 950.
(TIF)

Text S1 **Procedures to calculate the partial autocorrelation function for a time series.**
(DOCX)
