# Supplementary figures and images for: Step Detection in Single-Molecule Real Time Trajectories Embedded in Correlated Noise
Source: PLoS One. 2013 Mar 22;8(3):e59279. doi: 10.1371/journal.pone.0059279 (PMC3606409; doi:10.1371/journal.pone.0059279)

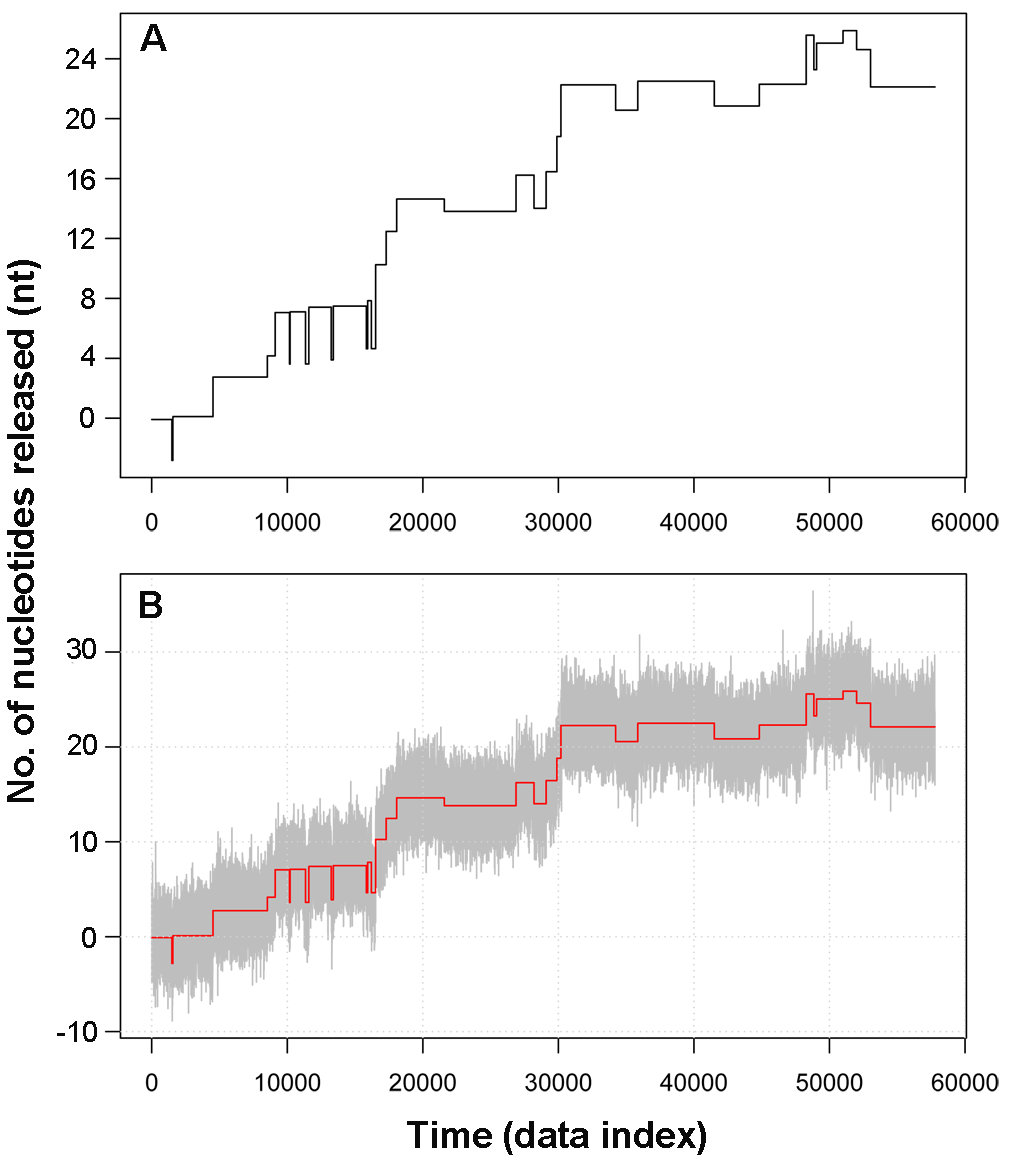

Supplement: Figure S1 — Simulated single-molecule RNA unwinding trajectory. Panel (A) shows the simulated step function, which indicates the true underlying steps; (B) shows one realization of simulated unwinding trace after addition of AR noise of order 7 on top of the step function shown in (A). The step function is shown in red, and the trajectory with noise is shown in grey. (TIF) [file pone.0059279.s001.tif]

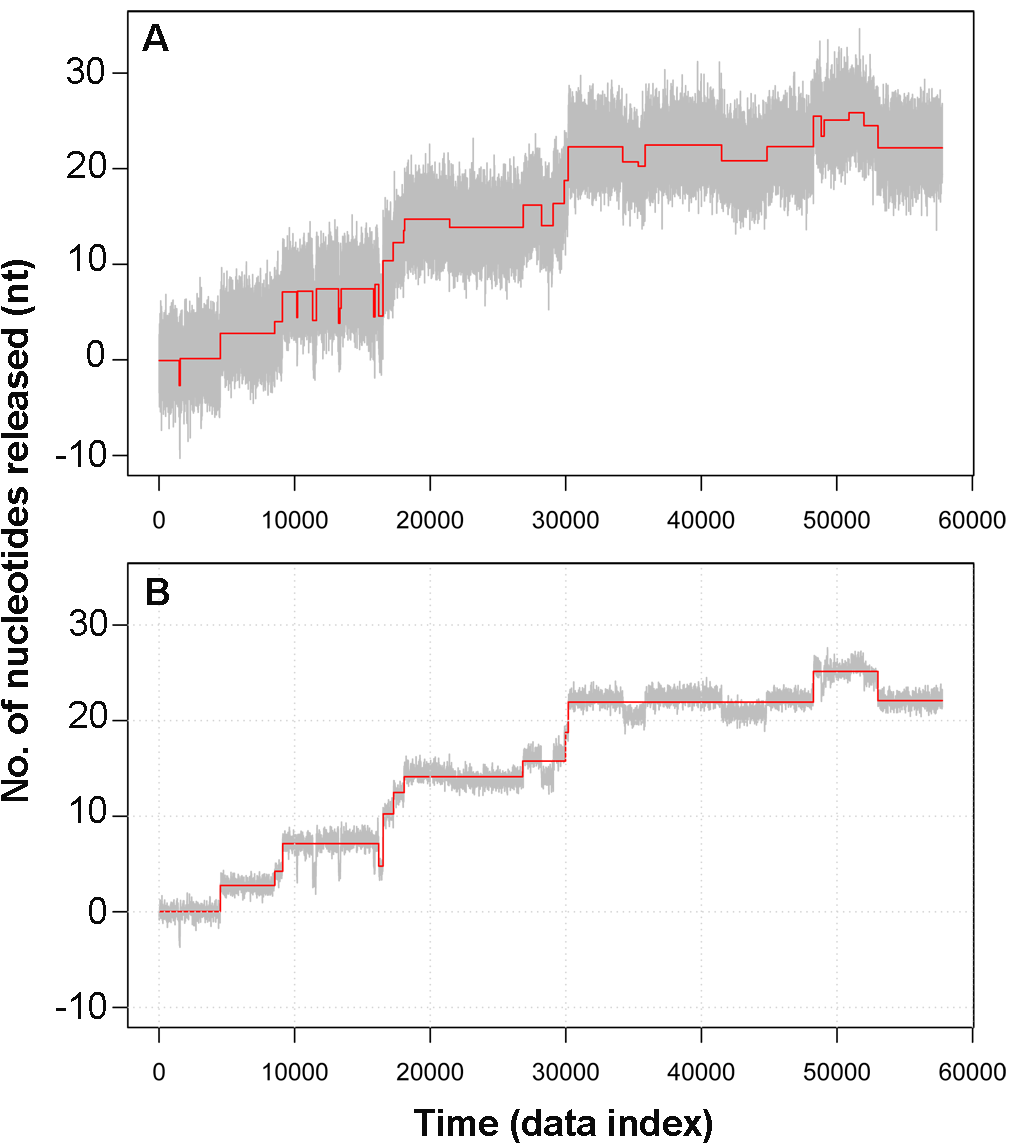

Supplement: Figure S2 — Representative best fits of simulated RNA unwinding traces from two different procedures. Panel (A) shows one of the best fits obtained from GLS method. The trajectory was simulated from the step function shown in Fig. S1A plus Gaussian white noise. The fit is in red, and the trajectory at 2.5 kHz is in grey. Panel (B) shows one of the best fits for simulated trajectories obtained from KERS method. The trajectory was simulated from the step function shown in Fig. S1A plus correlated noise of AR(7), and further filtered and decimated to 250 Hz using a boxcar filter. The fit is in red, and the trajectory at 250 Hz is in grey. (TIF) [file pone.0059279.s002.tif]

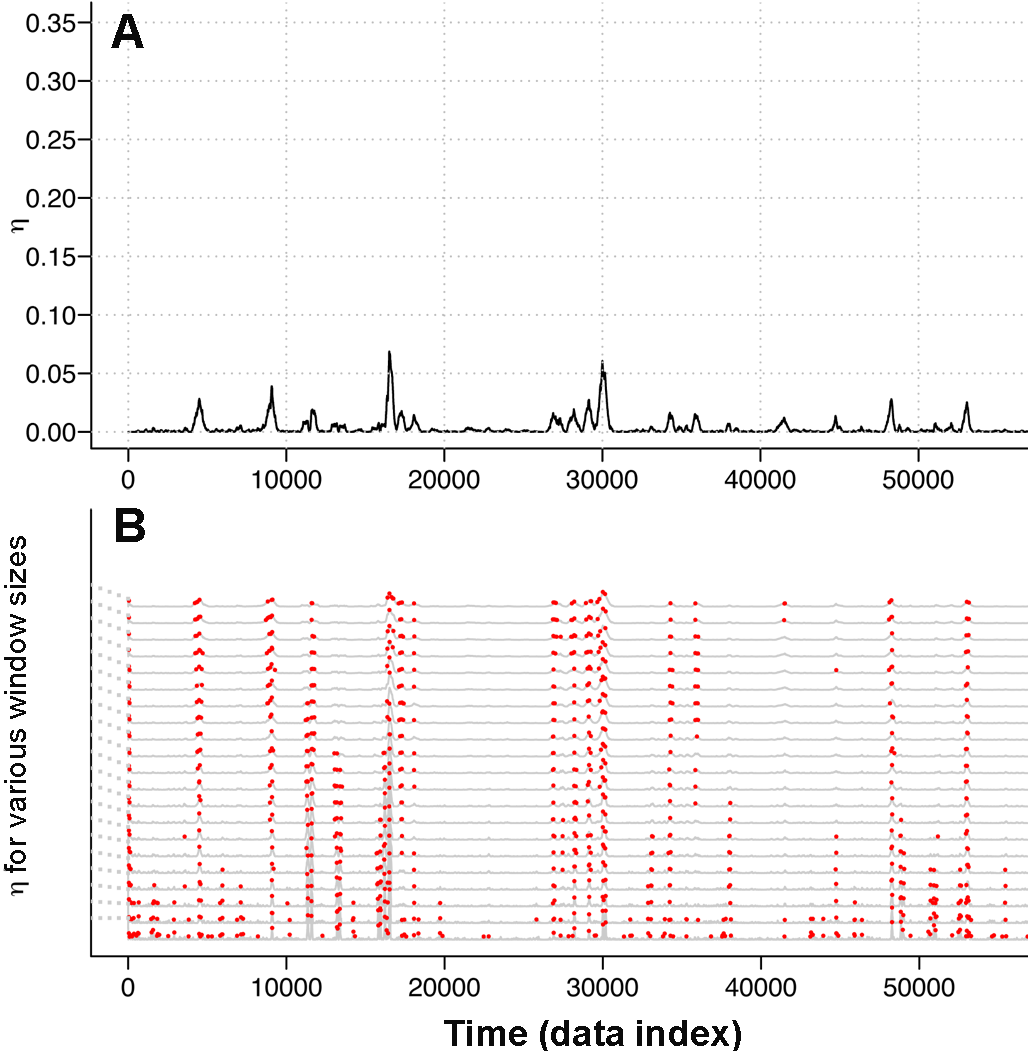

Supplement: Figure S3 — The statistic η computed for the simulated single-molecule trace in Fig. S1B. (A) from a window size of 500 and (B) shows a stack of η calculated using a set of window size, which includes 10, 30, 40, 50, 60, 70, 80, 90, 100, 125, 200, 275, 350, 425, 500, 575, 650, 725, 800, 875, and 950. (TIF) [file pone.0059279.s003.tif]
