# Supplementary material for: Step Detection in Single-Molecule Real Time Trajectories Embedded in Correlated Noise
Source: PLoS One. 2013 Mar 22;8(3):e59279. doi: 10.1371/journal.pone.0059279 (PMC3606409; doi:10.1371/journal.pone.0059279)
Supplement: Text S1 — Procedures to calculate the partial autocorrelation function for a time series. (DOCX) [file pone.0059279.s004.docx]

**Supporting Text S1**

**Partial Autocorrelation Function (PACF)**. To compute PACF for a time series at lag h, we regress data points h lags apart on the data points between them and compute the correlation between the resulting residuals in three steps as follows.

1. Consider the series $x_{t},\ldots,x_{T}$. (For lag h, t=h) . Regress these data points on the previous

h-1 data points, i.e.

This provides residuals $\varepsilon_{t},\ldots,\varepsilon_{T}$. Lets denote these residuals by RES1.

1. Next regress the points $x_{t-h},\ldots,x_{T-h}$on the succeeding h-1 data points, i.e.

This provides residuals $\varepsilon_{t-h},\ldots,\varepsilon_{T-h}$. Lets denote these residuals by RES2.

1. The PACF at lag h is the correlation between Residuals RES1 and RES2, i.e.

PACF(h) = Corr(RES1,RES2)

Consider an autoregressive series of order p and per Eq. 6, the data points can be adequately described by the previous p data points. Thus for data points that are p+1 lags apart, residuals (RES1) will have no further information but contain i.i.d Gaussian random noise. Similarly the other set of residuals (RES2) will be i.i.d Gaussian random noise and these two sets of residuals will be uncorrelated. This will hold true for lags greater than or equal to p+1. For lags equal to or less than p, the series is not adequately described by fewer than p past values, thus some information regarding the variation in the data is left in the residuals. These sets of residuals will tend to be correlated and thus give rise to a non-zero PACF.
